# Mitochondrial Metabolism and EV Cargo of Endothelial Cells Is Affected in Presence of EVs Derived from MSCs on Which HIF Is Activated

**DOI:** 10.3390/ijms24066002

**Published:** 2023-03-22

**Authors:** Federica Zanotti, Ilaria Zanolla, Martina Trentini, Elena Tiengo, Tommaso Pusceddu, Danilo Licastro, Margherita Degasperi, Sara Leo, Elena Tremoli, Letizia Ferroni, Barbara Zavan

**Affiliations:** 1 Translational Medicine Department, University of Ferrara, 44121 Ferrara, Italy; 2 Biomedicine Department, University of Ferrara, 44123 Ferrara, Italy; 3 AREA Science Park, Padriciano 99, 34149 Trieste, Italy; 4 GVM Care & Research, Maria Cecilia Hospital, 48033 Cotignola, Italy

**Keywords:** mesenchymal stem cells, endothelial cells, deferoxamine mesylate, miRNA, transcriptome

## Abstract

Small extracellular vesicles (sEVs) derived from mesenchymal stem cells (MSCs) have attracted growing interest as a possible novel therapeutic agent for the management of different cardiovascular diseases (CVDs). Hypoxia significantly enhances the secretion of angiogenic mediators from MSCs as well as sEVs. The iron-chelating deferoxamine mesylate (DFO) is a stabilizer of hypoxia-inducible factor 1 and consequently used as a substitute for environmental hypoxia. The improved regenerative potential of DFO-treated MSCs has been attributed to the increased release of angiogenic factors, but whether this effect is also mediated by the secreted sEVs has not yet been investigated. In this study, we treated adipose-derived stem cells (ASCs) with a nontoxic dose of DFO to harvest sEVs (DFO-sEVs). Human umbilical vein endothelial cells (HUVECs) treated with DFO-sEVs underwent mRNA sequencing and miRNA profiling of sEV cargo (HUVEC-sEVs). The transcriptomes revealed the upregulation of mitochondrial genes linked to oxidative phosphorylation. Functional enrichment analysis on miRNAs of HUVEC-sEVs showed a connection with the signaling pathways of cell proliferation and angiogenesis. In conclusion, mesenchymal cells treated with DFO release sEVs that induce in the recipient endothelial cells molecular pathways and biological processes strongly linked to proliferation and angiogenesis.

## 1. Introduction

Stem-cell-based therapies have recently gained popularity as a promising approach to support regenerative processes. Among mesenchymal stem cells (MSCs), adipose stem cells (ASCs) are very attractive due to the simple method of harvesting them and the remarkably high cell yield [1]. Nevertheless, some impediments still limit the clinical translation of cell-based therapies. For this reason, research is increasingly focused on the paracrine mediators, the small extracellular vesicles (sEVs), which work between MSCs and target cells. sEVs are vesicles of endosomal origin with sizes in the range of 30–150 nm that bring RNAs, proteins, and lipids to recipient cells and thus have an important role in intercellular communication [2,3]. sEVs have regenerative attributes like parenteral cells, and these may exceed the unwanted effects associated with stem cell transplantation. In fact, sEVs have a lower possibility of immune rejection, and they are more stable and storable [4,5]. sEVs have been isolated from numerous sources of MSCs, and their regenerative properties have been investigated. For instance, sEVs obtained from bone marrow showed a prevalent effect on cell proliferation and viability, sEVs from dental-pulp-derived MSCs showed distinct transcriptomic signatures of neurogenesis, while sEVs from adipose tissue showed a significantly improved ability to promote endothelial cell migration and angiogenesis [6,7]. Recently, MSC-derived sEVs have been attracting interest as a possible novel therapeutic agent for the management of different cardiovascular diseases (CVDs). Moreover, sEVs secreted by MSCs have shown cytoprotection, the stimulation of angiogenesis, and the modulation of macrophage infiltration in peripheral arterial diseases, atherosclerosis, and myocardial infarct [8,9,10].

In vivo, MSCs reside in niches in low-oxygen conditions, and conversely, in vitro culture conditions are often at atmospheric oxygen tensions [11]. However, it has been observed that hypoxic culture condition could significantly enhance the secretion of angiogenic mediators from MSCs [12,13]. Likewise, sEVs derived from MSCs preconditioned by hypoxia promote the angiogenesis, proliferation, and migration of endothelial cells in vivo and in vitro [14]. Moreover, the overexpression of the hypoxia-inducible factor 1α (HIF-1α) in MSCs improves angiogenesis in endothelial cells by the release of Jagged1-carrying sEVs [15]. Furthermore, iron-chelating deferoxamine mesylate (DFO) can be added to the culture medium as a useful substitute for environmental hypoxia. Various studies have proved that compared with hypoxia, the hypoxia mimetic agent could also induce related hypoxic genes. De facto, DFO is a prolyl-4 hydroxylase inhibitor that stabilizes HIF-1α under normoxic conditions through the inhibition of the prolyl hydroxylases enzyme, which targets the HIF-1 protein through degradation. Since hypoxic conditioning enhances the regenerative potential of ASCs through the upregulation of the transcription factor HIF-1α, the iron-chelating DFO can be used to mimic the increase in HIF-1α expression [16].

The improved regenerative potential of DFO-treated ASCs has been attributed to the increased release of angiogenic factors [17]; however, whether this effect is also mediated by the secreted sEVs has not yet been investigated. The aim of this study is to investigate the biological effects of sEVs released from DFO-treated ASCs (DFO-sEVs) on human umbilical vein endothelial cells (HUVECs). Transcriptome sequencing and miRNA profiling of the sEV cargo (sEV-miRNA) of treated HUVECs have shown improvements in mitochondrial oxidative phosphorylation, as well as signaling pathways related to cell proliferation and angiogenesis.

## 2. Results

### 2.1. DFO-sEVs Production and HUVEC Treatment

**Figure 1:**
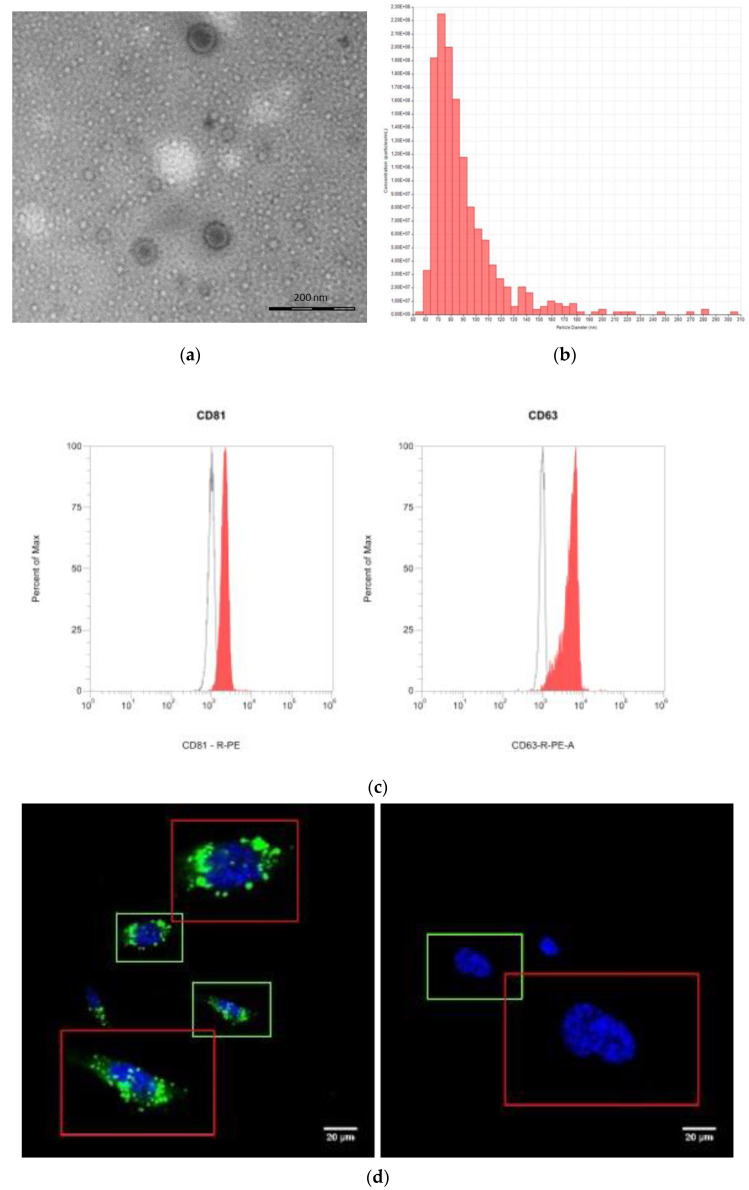
DFO-sEV production and HUVEC treatment. (**a**) Representative image of DFO-sEVs at TEM. sEVs appear with the typical bilayer cup-shaped membrane structure. (**b**) Particle size distribution and concentration of DFO-sEVs analyzed by tunable resistive pulse sensing: mean diameter of 90 ± 30.9 nm, mode of 73 nm, and particle size distribution of D10 67, D50 82, and D90 121. The average concentration was 1.33 × 10^9^ particles/mL. (**c**) Flow cytometry of DFO-sEVs showing positivity to surface markers: CD81 and CD63 (DFO-sEVs in red, vehicle in gray). (**d**) Representative image of the uptake of PKH67-labeled green fluorescent DFO-sEVs (left) and of negative control, i.e., PKH67-labeled PBS, (right) after 24 h of incubation. Nuclei stained with Hoechst 33342 (blue). In red area, 2× magnification of adjacent green area.

Human ASCs were treated with 100 μM DFO to stabilize HIF-1 under normoxic conditions [18,19]. In particular, commercially available hASCs were seeded in a six-well plate at a density of 4 × 10^5^ hASCs/well in a complete medium. The following day, cells were washed twice with phosphate-buffered saline (PBS, EuroClone) and then incubated with 100 μM deferoxamine mesylate (DFO; Thermo Fisher Scientific, Waltham, MA, USA) in EV-depleted DMEM overnight. After the overnight treatment, the conditional medium was harvested and the DFO-sEVs were isolated through a technology based on Norgen’s proprietary resin, which allows the purification of intact extracellular vesicles [20]. The DFO-sEVs appeared like rounded structures below 100 nm in the transmission electron microscopy (TEM) (Figure 1a). Tunable resistive pulse sensing (RPS) analysis confirmed the dimension of the vesicles: mean diameter of 90 ± 30.9 nm and mode of 73 nm. The particle size distribution of D10, D50, and D90 was 67 nm, 82 nm, and 121 nm, respectively. The average concentration was 1.33 × 10^9^ particles/mL (Figure 1b). Moreover, the DFO-SEVs were positive for superficial markers CD81 and CD63, as shown in Figure 1c.

Purified DFO-sEVs were added to HUVEC cultures for 24 h, and sEV internalization was detected through observation under confocal microscopy. In Figure 1d, HUVECs incubated with PKH67 green fluorescent DFO-sEVs (left) are compared with cells incubated with the negative control (right), i.e., probe-labeled PBS. The red areas show a 2× magnification of the green areas. After remaining for 72 h in an EV-depleted medium, the total RNA was isolated from HUVECs and the conditioned medium was harvested for sEV (HUVEC-sEVs) recovery.

### 2.2. RNA Sequencing of HUVECs Treated with DFO-sEVs

**Figure 2:**
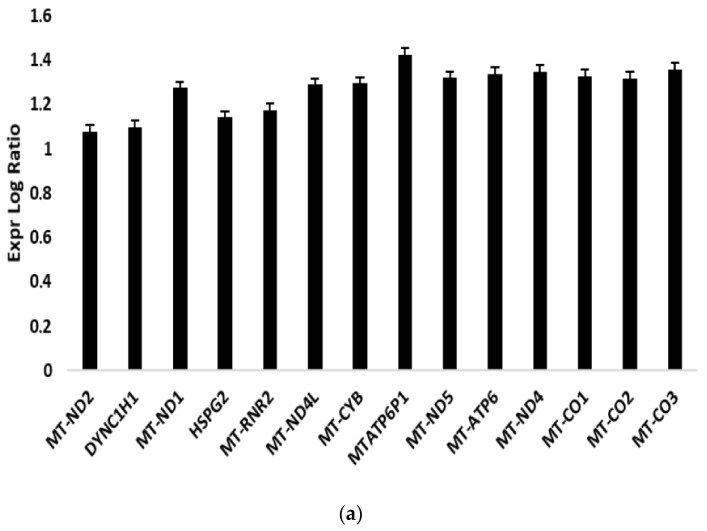

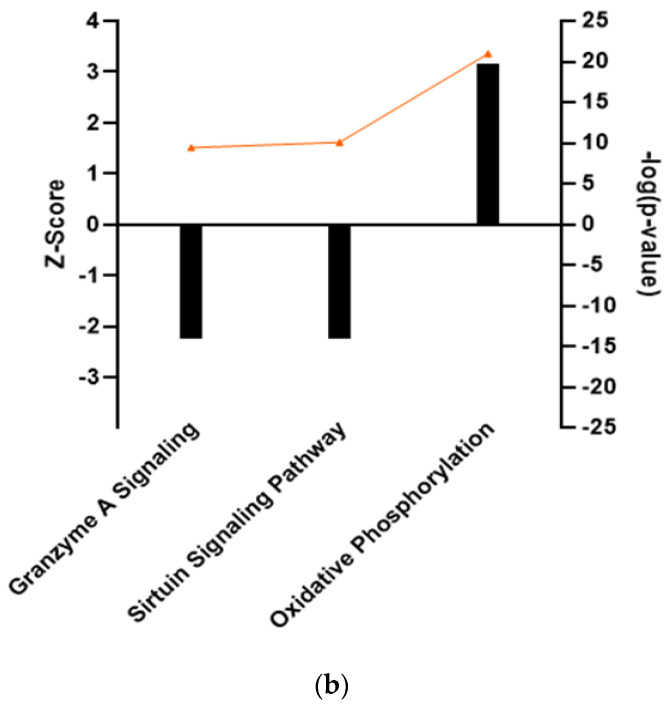
Ingenuity pathway analysis on HUVECs treated with DFO-sEVs. (**a**) Expression of logarithmic ratios of differentially regulated genes for treated HUVECs compared to untreated HUVECs. (**b**) Canonical pathway analysis by IPA: predicted state of activation/inhibition is displayed as Z-Score (black bars), and significance is expressed as −log10 (*p*-value) (orange line).

Total RNA extracted from DFO-sEV-treated HUVECs and untreated HUVECs were sequenced, and ingenuity pathway analysis (IPA) was performed on differentially expressed genes (DEGs). Fourteen genes were significantly upregulated in HUVECs treated with DFO-sEVs (Figure 2a), including genes encoding mitochondrially encoded NADH dehydrogenase (MT-ND1, MT-ND2, MT-ND4, MT-ND5, and MT-ND4L), mitochondrially encoded cytochrome c oxidase (MT-CO1, MT-CO2, and MT-CO3), mitochondrially encoded cytochrome b (MT-CYB), mitochondrially encoded ATP synthase 6 (MT-ATP6), dynein cytoplasmic 1 heavy chain 1 (DYNC1H1), and heparan sulfate proteoglycan 2 (HSPG2). IPA canonical pathways analysis revealed that these genes are associated with three biological pathways: granzyme A signaling, the sirtuin signaling pathway, and oxidative phosphorylation (Figure 2b). The granzyme A and sirtuin pathways were significantly downregulated in HUVECs treated with DFO-sEVs (Z-score of −2.236; −log (*p*-value) of 9.43 and 10.1, respectively); on the contrary, oxidative phosphorylation was significantly upregulated (Z-score of +3.162; −log (*p*-value) of 21).

**Figure 3:**
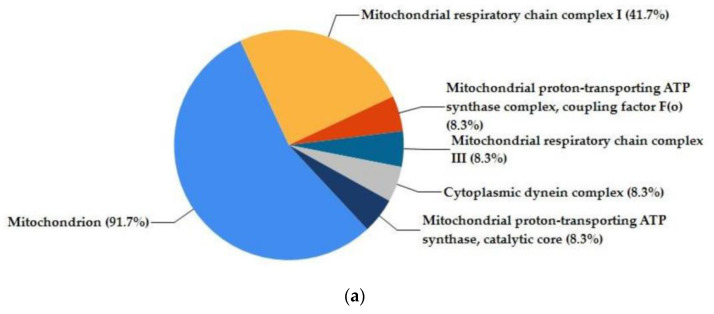

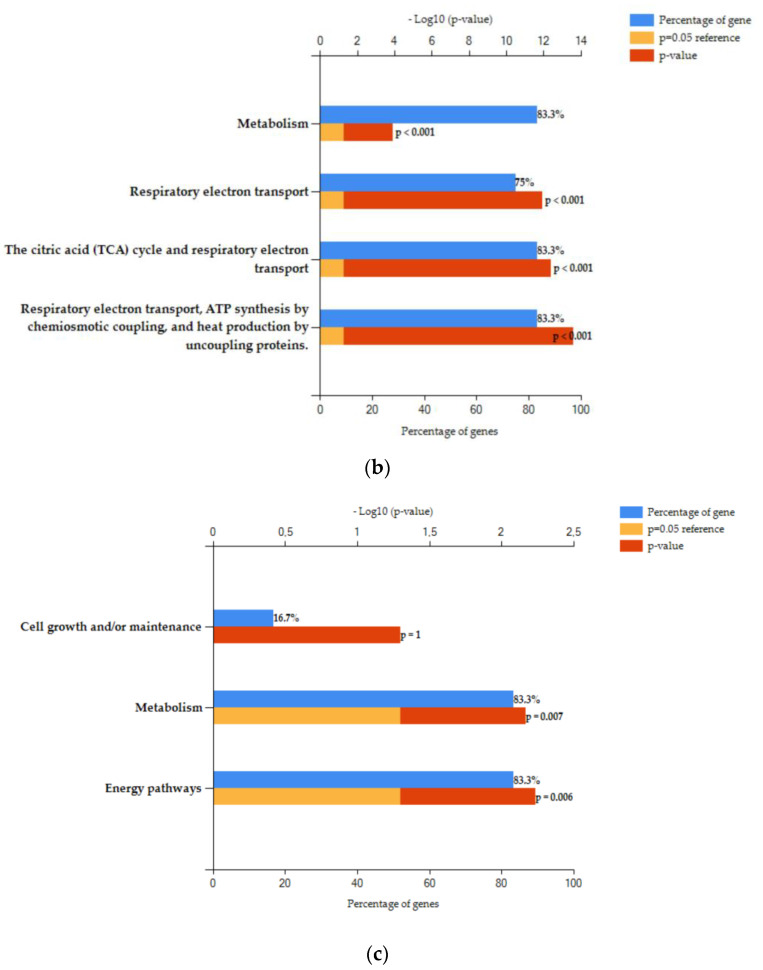
Functional enrichment analysis with FunRich software version 3. (**a**) Pie chart of cellular component enrichment. (**b**) Biological pathway enrichment. (**c**) Biological process enrichment. Percentage of genes involved in each function (blue bar), *p*-value (red bar), and reference (orange bar).

**Figure 4:**
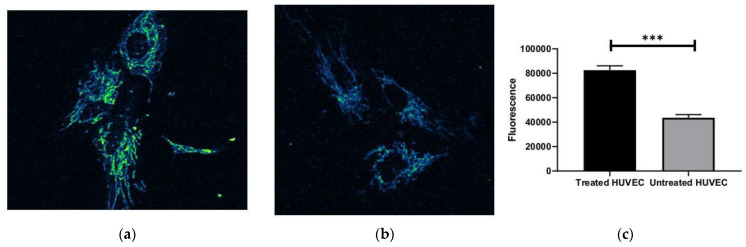
Mitochondrial membrane potential of HUVECs treated with DFO-sEVs compared to untreated cells. Representative images of (**a**) treated and (**b**) untreated HUVECs. (**c**) Fluorescence intensity quantification with ImageJ software vs8. *** *p*-value < 0.001.

A functional enrichment analysis of the 14 significant genes was performed using the FunRich database for the categories cellular component, biological pathway, and biological process (Figure 3). The significant upregulated genes were mostly mitochondrial components, as shown in the pie chart (Figure 3a). The enriched biological pathways were respiratory electron transport, ATP synthesis by chemiosmotic coupling, heat production by uncoupling proteins, the citric acid cycle and respiratory electron transport, the respiratory electron transport, and metabolism (Figure 3b). Three biological processes resulted in enriched cell growth and maintenance, metabolism, and energy (Figure 3c). Moreover, HUVECs treated with DFO-sEVs showed amplified mitochondrial membrane potential compared to untreated cells, as displayed by the microscopy analysis with a fluorescent probe that accumulates in the mitochondria in a membrane-potential-dependent manner (Figure 4).

### 2.3. sEV-miRNA Expression Profiling of HUVECs Treated with DFO-sEVs

**Figure 5:**
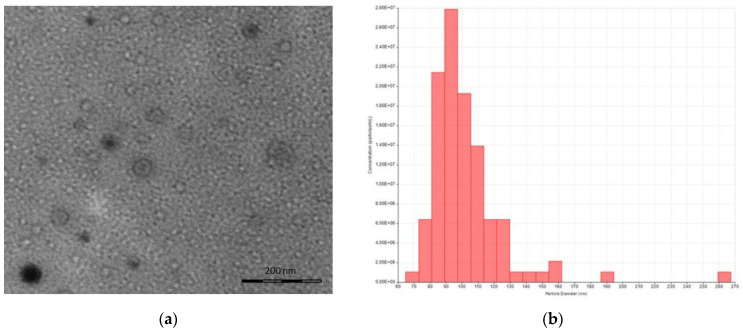
HUVEC-sEV isolation. (**a**) Representative TEM image. (**b**) Size distribution and concentration using tunable resistive pulse sensing instrument qNano (iZON Science, Oxford, UK). Average hydrodynamic diameter was 102 ± 24.9 nm, mode 93 nm, d10 83 nm, d50 97 nm, d90 123 nm. Average concentration was 2.21 × 10^7^ particles/mL.

HUVEC-sEVs were isolated as previously described [20]. The TEM image and tunable resistive pulse sensing analysis showed the distinctive features of sEVs: bilayer cup-shaped membrane structure as a result of dehydration during sample preparation (Figure 5a), and particle size dimension roughly of 70–130 nm (Figure 5b).

**Figure 6:**
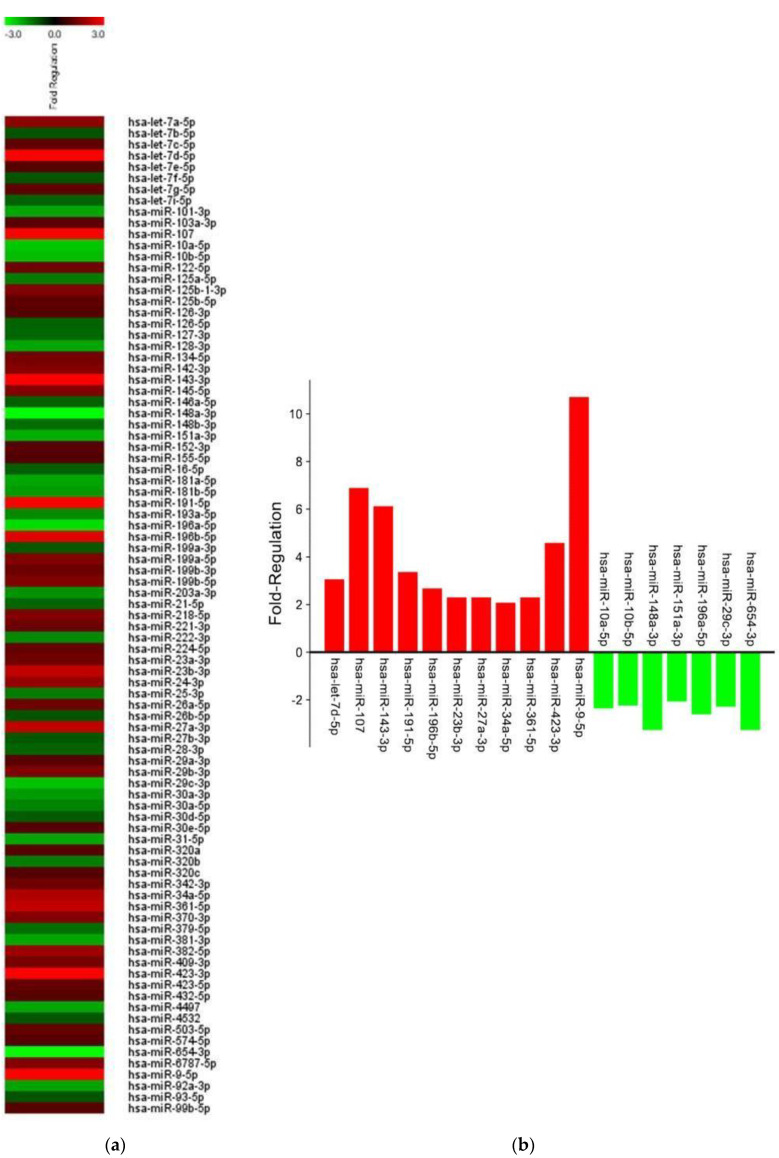
sEV-miRNA sequencing of HUVECs treated with DFO-sEVs. (**a**) Fold regulation of 89 identified miRNAs (upregulation cut-off > 2; downregulation cut-off < −2). (**b**) Histogram with 11 upregulated and 7 downregulated miRNAs.

**Figure 7:**
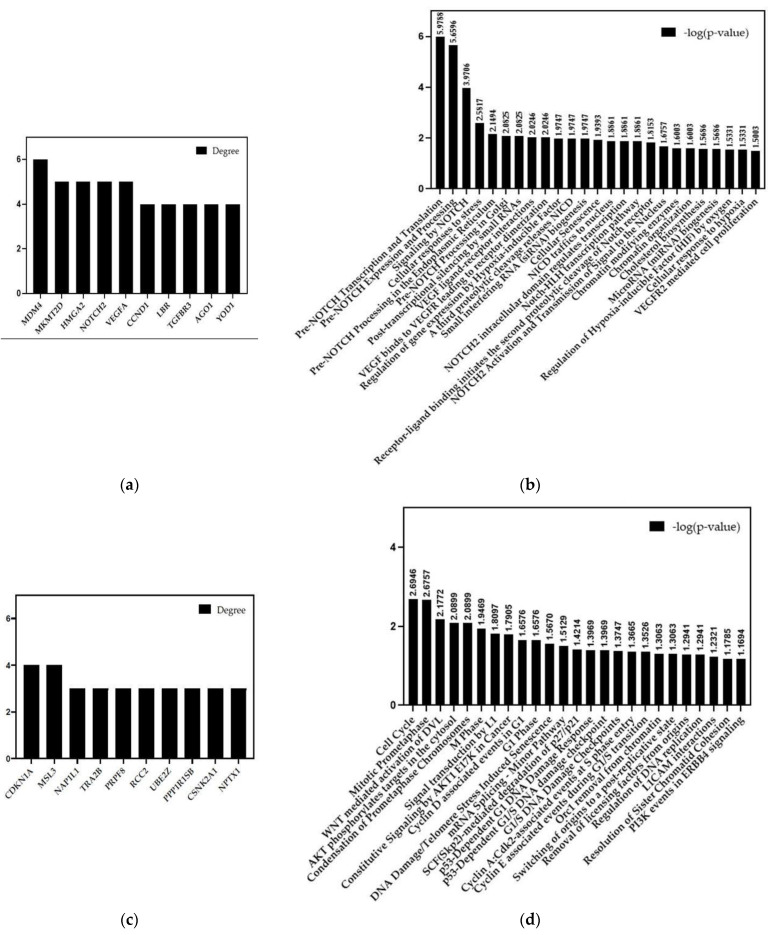
Functional enrichment analysis of sEV-miRNA from treated HUVECs. (**a**) The 10 main target genes regulated by the upregulated sEV-miRNAs. (**b**) Biological pathways regulated by these target genes. (**c**) The 10 main target genes regulated by the downregulated sEV-miRNAs. (**d**) Biological pathways regulated by these target genes.

Illumina sequencing and IPA analysis were performed on sEV-miRNAs of treated HUVECs. Among the identified 89 miRNAs (Figure 6a), 18 miRNAs showed a significant fold-regulation value (upregulation cut-off > 2; down regulation cut-off < −2) (Figure 6b). Precisely, 11 miRNAs resulted in upregulation, while 7 resulted in downregulation. The functional enrichment analysis of upregulated and downregulated miRNAs was performed using the miRNet software Vs2 and Reactome biological pathway database (Figure 7). Figure 7a shows the 10 main target genes of the 11 upregulated miRNAs, such as MDM4, NOTCH2, VEGFA, CCND1, and TGFBR3. These target genes encoding proteins and receptors are related to several biological pathways of NOTCH, VEGFR, and HIF (Figure 7b). The ten main target genes of the seven downregulated miRNAs (Figure 7c) were related to cell proliferation and cell cycle progression (Figure 7d).

## 3. Discussion

sEVs derived from MSCs grown under hypoxic conditions can induce desirable biological effects on receiver cells, such as improvements in proliferation and migration [14]. These effects appear to be related to hypoxia, which induces HIF-1α mRNA expression via the PI3K/AKT pathway and the activation of NFκB [21]. Gonzalez-King and colleagues reported that the overexpression of HIF-1α in dental-pulp-derived MSCs improves angiogenesis in endothelial cells by the release of Jagged1-carrying exosomes [15]. In the present study, instead, ASCs were treated with the iron-chelating DFO to induce an increase in the HIF-1 expression. Basically, DFO stabilizes HIF-1 under normoxic conditions through the inhibition of the prolyl hydroxylases enzyme, which targets HIF-1 protein through degradation [16]. Therefore, the increase in HIF-1α expression was not induced through gene overexpression, nor through the maintenance of the mesenchymal cells in hypoxic conditions, but through the treatment with a molecule that stabilizes HIF-1 protein by blocking its degradation. After overnight treatment with DFO, the conditioned culture medium was collected to isolate sEVs by precipitation. Single-particle characterization and quantitation were performed, including imaging by electron microscopy, particle tracking techniques, and flow cytometry, following the guidelines of Minimal Information for Studies of Extracellular Vesicles [22] (Figure 1). With TEM, EVs appeared with a diameter of less than 100 nm and with the typical bilayer cup-shaped membrane structure, due to dehydration during sample preparation [23]. Particle size, particle size distribution, and particle concentration were measured through tunable resistive pulse sensing (RPS) with a qNano device. Specifically, the particle mean diameter was 90 nm and the mode was 73 nm. The particle size distribution of D10, D50, and D90 was 67 nm, 82 nm, and 121 nm, respectively. Previously, Connor et al. [24] analyzed EVs with a qNano device, defining the particles with the average size of 92 nm as small EVs. Moreover, the presence of sEV markers was analyzed by the classical flow cytometry of bead-captured EVs [25]. Polystyrene beads (4.5 μm diameter) coated with a primary monoclonal antibody specific for the CD63 or CD81 membrane antigen were incubated overnight with DFO-sEVs. Then, the bead-bound sEVs were stained with a fluorescent-conjugated antibody for CD63 or CD81. Overall, the particles isolated from DFO-treated MSCs could be defined as small EVs because they were bilipid membrane vesicles with a mean diameter of 90 nm and positive to sEV markers CD81 and CD63 (Figure 1). DFO-sEVs were used to treat the recipient endothelial cells prior to transcriptome sequencing and SEV-miRNA profiling (Figure 1d). The transcriptome of HUVECs treated with DFO-sEVs was analyzed through IPA. Fourteen genes were significantly upregulated. Among the upregulated genes, 12 were mitochondrial genes related to the oxidative phosphorylation, including MT-ND2, MT-ND1, MT-RNR2, MT-ND4L, MT-CYB, MT-ND5, MT-ATP6, MT-ND4, MT-CO1, MT-CO3, MT-CO2, and MTATP6P1. The last two genes were DYNC1H1 and HSPG2 (Figure 2a). DYNC1H1 encodes cytoplasmic dynein that acts as a motor for the intracellular retrograde motility of vesicles and organelles along microtubules. It is reported that the loss of function of this gene causes a significant decrease in cell viability and cell proliferative ability [26]. The HSPG2 gene encodes “Perlecan”, the proteoglycan key component of the vascular extracellular matrix, which is able to maintain the endothelial barrier function [27]. Overall, the expression profile of DFO-sEV-treated HUVECs shows enhanced mitochondrial activity and the overexpression of proactive genes that could lead to the proliferation, development, and preservation of the extracellular matrix integrity. IPA was performed to identify the canonical pathways that are most significant to the transcriptome sequencing outcome and to categorize upregulated genes. Three canonical pathways resulted in significant predictions: the granzyme A signaling and sirtuin signaling pathway resulted in an inactivated profile; in contrast, oxidative phosphorylation showed an activated profile (Figure 2b). Granzyme A signaling induces a caspase-independent cell death pathway [28], whereas sirtuin signaling can induce aging [29]. Oxidative phosphorylation is the metabolic process that leads to ATP production inside cells [30]. The canonical pathways identified by IPA highlighted a possible positive effect of DFO-sEVs on HUVECs, as they increased the expression of genes involved in oxidative phosphorylation and therefore the production of energy; however, the treatment with DFO-sEVs reduced the expression of genes connected to cell death and aging. These observations were proved through functional analysis using the FunRich software and through mitochondrial membrane potential detection. The FunRich enrichment showed improvement in mitochondrial respiratory electron transport and ATP production, cell growth, and maintenance (Figure 3). Therefore, the canonical pathway of IPA and the FunRich analysis agreed in the identification of the same pathway, namely, mitochondrial respiration. Furthermore, the mitochondrial membrane potential was assessed with a probe that accumulates in the mitochondria in a membrane-potential-dependent manner. The microscopy analysis confirmed that the mitochondrial increased activation for DFO-sEV-treated HUVECs compared to untreated cells (Figure 4).

The data on HUVEC responses to DFO-sEVs were implemented by sequencing the miRNA content in sEVs derived from the treated endothelial cells. The IPA on sEV-miRNAs revealed 11 upregulated and 7 downregulated miRNAs in the treated HUVECs compared to untreated cells (Figure 6). The eleven upregulated miRNAs (hsa-let-7d-5p, hsa-mir-107, hsa-mir-143-3p, hsa-mir-191-5p, hsa-mir-196b-5p, hsa-mir-23b-3p, hsa-mir-27a-3p, hsa-mir-34a-5p, hsa-mir-361-5p, hsa-mir-423-3p, and hsa-mir-9-5p) have pro-angiogenesis and pro-proliferative action on cells, as reported in the literature. For instance, mir-9-5p can induce enhancement in angiogenesis, proliferation, and migration and prevent apoptosis in endothelial progenitor cells [31]. The upregulation of mir-107, mir-143-3p, mir-23b-3p, and mir-27a-3p has also been reported to have a pro-angiogenic function [32,33,34]. The let-7d-5p and mir-196b-5p miRNAs have proliferation-inducing functions [35,36]. In contrast, the seven downregulated miRNAs (hsa-miR-10a-5p, hsa-miR-10b-5p, hsa-miR-148a-3p, hsa-miR-151a-3p, hsa-miR-196a-5p, hsa-miR-29c-3p, and hsa-miR-654-3p), if expressed, have anti-proliferative, anti-angiogenetic, and pro-inflammatory action [37,38,39,40,41,42]. It can be assumed that the downregulation of these miRNAs in HUVEC-sEVs serves to promote proliferation and prevent inflammation in the recipient cells. The enrichment analysis of the significantly upregulated and downregulated miRNAs was performed using the miRNet software (Figure 7). The functional enrichment of the ten main target genes of upregulated miRNAs with the Reactome database highlighted a link with the NOTCH, VEGFR, and HIF signaling pathways, namely, pathways related to proliferation and angiogenesis [43]. The functional enrichment on the ten main target genes of downregulated miRNAs revealed enrichment of the biological pathways that control the cell cycle progression and AKT/PI3K signaling [44].

In the present in vitro study, the effects of DFO on ADSCs were investigated, with a particular focus on the messages conveyed by the sEVs released after the treatment. In particular, human endothelial cells were incubated with sEVs derived from DFO-treated mesenchymal cells to evaluate the alteration in cellular transcriptomes and miRNA cargo in sEVs. The transcriptomic analysis revealed the downregulation of genes connected to cell death and aging and the upregulation of mitochondrial genes linked to oxidative phosphorylation. The potentiating effect of the treatment with DFO-sEVs on endothelial cell mitochondria was also highlighted by an increase in the mitochondrial membrane potential. Moreover, the functional enrichment analysis of the sEV-miRNAs released from the endothelial cells showed a connection with the signaling pathways related to cell proliferation and angiogenesis.

In conclusion, adipose-derived mesenchymal cells treated with iron-chelating deferoxamine release small extracellular vesicles that induce in the recipient endothelial cells molecular pathways and biological processes strongly linked to energy storage, proliferation, and angiogenesis.

## 4. Materials and Methods

### 4.1. Adipose Stem Cell Treatment and SEV Characterization

Human adipose stem cells (ASCs; purchased from ScienCell Research Laboratories, Inc., Carlsbad, CA, USA) were maintained in Dulbecco’s Modified Eagle’s Medium (DMEM, EuroClone, Milano, Italy) supplemented with 10% fetal bovine serum (FBS, EuroClone, Milano, Italy) and 1% Antibiotic–Antimycotic (Thermo Fisher Scientific, Waltham, MA, USA) until the experiments were conducted. In a 6-well plate, 4 × 10^5^ hASCs/well (passages between 2 and 4) were seeded in the complete medium. The following day, the cells were washed twice with phosphate-buffered saline (PBS, EuroClone) and then incubated with 100 μM deferoxamine mesylate (DFO; Thermo Fisher Scientific, Waltham, MA, USA) in EV-depleted DMEM overnight.

The sEVs (DFO-sEVs) were isolated from the conditioned medium of DFO-treated hASCs using a Cell Culture Media Exosome Purification Kit (Norgen Biotek Corp., Thorold, ON, Canada) according to the manufacturer’s instructions.

For transmission electron microscopy (TEM), the sEVs were fixed in a 2% glutaraldehyde solution in phosphate buffer (ratio 1:1). The sEVs were then deposited, rinsed, and stained with heavy metal compounds onto a gridded slide according to the standard protocols. The slide was visualized with a TEM Zeiss EM 910 instrument (Zeiss, Oberkochen, Germany).

The distribution size and diameter of the sEVs were analyzed with a qNano platform (iZON Science, Oxford, UK). The analyses were performed with NP150 nanopores and CPC200 calibration particles at 20 mbar pressure. The results were analyzed with the Izon control suite v3.4.

For flow cytometry, CD81-positive and CD63-positive sEVs were isolated with Exosome-Human CD81 Flow Detection (Thermo Fisher Scientific, Waltham, MA, USA) and Exosome-Human CD63 Isolation/Detection Reagent (Thermo Fisher Scientific, Waltham, MA, USA), respectively. Briefly, 100 μL of an sEV suspension was incubated with 20 μL of CD81 or CD63 magnetic beads at 4 °C overnight. Bead-bound sEVs were washed twice with an Assay Buffer (0.1% BSA in PBS), and then labeled with 20 μL of mouse anti-human CD81-PE monoclonal antibody (BD Pharmingen™, BD Biosciences, San Jose, CA, USA) or 5 μL of mouse anti-human CD63-PE monoclonal antibody (eBioscience, San Diego, CA, USA). After incubation in an orbital shaker at 1000 rpm for 1 h, the bead-bound sEVs were washed twice with an Assay Buffer. Negative control was performed by staining PBS (vehicle) instead of the sEVs. Flow cytometric detection was performed with an Attune™ N × T Acoustic Focusing Cytometer (Life Technologies, Carlsbad, CA, USA), and the data were analyzed with the Attune N × T Software version 2.5 (Life Technologies).

### 4.2. Endothelial Cell Treatment and Analyses

Human umbilical vein endothelial cells (HUVECs; Thermo Fisher Scientific, MA, USA) were cultured in an EBM^TM^-2 basal medium (Lonza, Basel, Switzerland) completed with EGM^TM^-2 SingleQuots^TM^ Supplements (Lonza). Into 6-well plates, HUVECs (passages between 2 and 4) at 2 × 10^5^ cells/well were seeded. After 24 h, 500 μL of DFO-sEVs at a concentration of 1.33 × 10^9^ particles/mL or an equal volume of PBS was added to the culture medium for 24 h. After 72 h of resting, the total RNA was isolated from the HUVECs using the Total RNA purification Plus kit (Norgen Biotek, Thorold, ON, Canada) according to the manufacturer’s instructions. sEV-miRNA was isolated from the conditional medium using Cell Culture Media Exosome Purification and an RNA Isolation Mini Kit (Norgen Biotek, Thorold, ON, Canada) according to the manufacturer’s instructions.

For internalization detection, the DFO-sEVs were stained with PKH67 (PKH67 Green Fluorescent, Sigma-Aldrich) for 20 min at 37 °C. An equal volume of PBS without sEVs was labeled with the green fluorescent probe and used as the negative control. Excess unincorporated dye was removed from the labeled solutions by using Exosome Spin Columns (MW 3000) (Thermo Fisher Scientific), following the manufacturer’s instructions. Then, HUVECs were incubated with the labeled sEVs for 24 h. After washing with PBS, the nuclei were stained with Hoechst 33342 (ThermoFisher Scientific) for 10 min. The cells were observed with a laser scanning confocal microscopy system (Nikon A1 confocal microscope, Nikon Corporation, Tokyo, Japan) equipped with a 60× objective. The zoomed-in insets in Figure 1d were produced with Fiji Vs4 software.

For the mitochondrial membrane potential, the cells were incubated with MitoTracker Red CMXRos (Thermo Fisher Scientific) for 30 min at 37 °C. After washing, the cells were immediately observed on a Nikon LiveScan Swept Field Confocal Microscope (SFC) Eclipse Ti equipped with NIS-Elements microscope imaging software and on a confocal laser scanning Olympus FV3000 microscope both equipped with a 63X oil immersion objective (N.A. 1.4). The red signal colocalization rate was evaluated using the JACOP colocalization counter available in the Fiji software (ImageJ). For each condition, the signal was also determined by manually counting the fluorescent puncta. For each ROI, the Manders’ parameter was calculated. For each condition, five replicates were observed, and four measurements were performed on each replicate.

### 4.3. Sequencing and Data Analysis

mRNA sequencing and miRNA profiling were carried out by Area Science Park (ASP, Trieste, Italy) with Illumina sequencing.

The total RNA was evaluated using NanoDrop 2000 (Thermo Fisher Scientific, Waltham, MA, USA) and Agilent Bioanalyzer 2100 (Agilent, Santa Clara, CA, USA). Libraries were created with 1 μg of the total RNA with the TruSeq Sample Preparation RNA Kit (Illumina Inc., San Diego, CA, USA) according to the manufacturer’s protocol. All libraries were quantified with the Qubit dsDNA BR Assay Kit (Thermo Fisher Scientific, Waltham, MA, USA) on a Qubit 2.0 Fluorometer (Thermo Fisher Scientific, Waltham, MA, USA). RNA sequencing was realized on a Novaseq 6000 sequencer (Illumina Inc., San Diego, CA, USA) according to the manufacturer’s protocol. FASTQ files were output with the Illumina BCLFASTQ v2.20 software. All raw files’ quality was verified with FASTQC software V4 (http://www.bioinformatics.bbsrc.ac.uk/projects/fastqc; accessed on 15 October 2022), and low-quality sequences were discarded from the analysis. Selected reads were aligned onto the complete human genome using Splices Transcripts Alignment to the Reference algorithm STAR version 2.7.3 using hg38 Genome Assembly and Genecode.v35 as the gene definition. The resulting mapped reads were included as the input for the feature count functions of the Rsubread packages and were used as gene counts for differential expression analysis using the Deseq2 package. Reads comparison was performed between DFO-sEV-treated HUVECs and untreated HUVECs. Differentially expressed genes (DEGs) were selected for log 2 (FR) < −1 or >1 and *p*-value < 0.05.

MiRNA-Seq libraries were prepared using the QIAseq miRNA Library Kit (QIAGEN; Hilden, Germany) and sequenced using Novaseq 6000 (Illumina; San Diego, CA, USA) in the 2 × 150 paired-end mode. Identification of miRNAs in the samples was performed using the QIAseq miRNA-NGS data analysis software V5 considering Single Read as the read type and Read 1 Cycles 75 as the read cycles.

All the datasets from RNA sequencing and miRNA sequencing were analyzed with the Qiagen Ingenuity Pathway Analysis (IPA) software. For the RNA sequencing analysis, IPA categorized all DEGs in canonical pathways. IPA can make a prediction on possible diseases and functions, which were ranked based on their significance (*p*-value) and predicted state of activation/inhibition (z-Score). The Z-score value was set with cut-off < −2 or >+2. RNA sequencing was used to perform functional, biological pathway, biological process, and cellular component enrichment with the FunRich software [45], while MiRNet was used to analyze functional enrichment for miRNA sequencing. miRNet provided miRNA target gene data that were collected from four well-annotated miRTareBase v8.0 databases. Functional enrichment with miRNet was performed on the Reactome Biological Pathway database [46]. All miRNet enrichment was reported with a Prism 8.03 software graphical view (GraphPad Software Inc., Boston, MA, USA).

The data are expressed as means ± SEM. Student’s *t*-test was used for comparing single comparisons. For multiple comparisons, one-way analysis of variance (ANOVA) was performed. A value of *p* < 0.05 was used as the benchmark for statistical significance.
